# The Curious Case of the Black Buboes

**DOI:** 10.7759/cureus.32495

**Published:** 2022-12-13

**Authors:** Arunava Saha, Shihla Shireen Kanamgode, Shari Mitra

**Affiliations:** 1 Internal Medicine, Saint Vincent Hospital, Worcester, USA; 2 ENT, Government Medical College and New Civil Hospital, Surat, IND

**Keywords:** malignancy mimic, hypermetabolic lymph nodes, mediastinal lymphadenopathy, nodal anthracosis, rare cause of diffuse lymphadenopathy, anthracosis

## Abstract

Anthracosis is an environmental lung disease caused by carbon deposition and pigmentation in the airways. However, in rare instances, it can also have systemic involvement. We present a patient with B-symptoms and diffuse lymphadenopathy who was diagnosed with the infrequently described nodal anthracosis. A 64-year-old Vietnamese gentleman with a 50-pack-year smoking history who was recently diagnosed with prostate cancer post-radical prostatectomy and awaiting radiation therapy presented with generalized weakness, low-grade fever, night sweats, and unquantifiable weight loss for a month. He was hemodynamically stable, and examination revealed bilateral inguinal and axillary lymphadenopathy. Computed tomography (CT) showed diffuse lymphadenopathy involving the mediastinum, hilar, axillary, mesenteric, retroperitoneal, and bilateral iliac chains with multiple diffuse pulmonary nodules. Laboratories disclosed anemia, thrombocytopenia, elevated erythrocyte sedimentation rate (ESR) and C-reactive protein (CRP), albumin-globulin (A-G) reversal, and sterile blood cultures. The disseminated intravascular coagulation panel was negative with normal fibrinogen and mildly elevated D-dimer. Autoimmune workup, including antinuclear antibody (ANA), was negative. Infectious workup included *Babesia*, *Ehrlichia*, *Anaplasma*, Lyme serology, QuantiFERON-TB Gold, HIV, and hepatitis panel, and all were negative. He was managed with broad-spectrum antibiotics, which were discontinued after a negative infectious workup. He also complained of a new-onset holocranial headache with no features of meningitis; an MRI with contrast revealed focal occipital leptomeningeal involvement and cerebral edema with occipital lymphadenopathy. A lumbar puncture was planned but deferred at the patient's request. An excisional lymph node biopsy of the left axillary lymph node revealed reactive follicular hyperplasia with no evidence of malignancy, with flow cytometry negative for any evidence of B- or T-cell malignancies. He continued to have persistent low-grade fevers. A bone marrow biopsy showed 70% cellularity with paratrabecular interstitial lymphoid aggregates composed of both T and B cells, which was nonspecific, and flow cytometry could not be done due to dry tap. An F-18-fluorodeoxyglucose positron emission tomography (FDG PET) scan showed extensive hypermetabolic disease both above and below the diaphragm with bulky mediastinal adenopathy and splenomegaly. Subsequently, he underwent a mediastinoscopy and biopsy of the mediastinal lymph nodes, which demonstrated reactive hyperplasia and abundant anthracitic pigment on microscopic examination, consistent with the diagnosis of nodal anthracosis. He was managed conservatively, discharged, and found to have spontaneously resolved symptoms at a six-week follow-up. Nodal anthracosis with PET-positive mediastinal and hilar lymphadenopathy is a rare presentation of anthracosis that mimics infectious conditions, granulomatous diseases, and malignancies. The pigment deposition can cause persistent inflammatory activity and should be considered an infrequent but important explanation of lymphadenopathy in patients without known biomass exposure.

## Introduction

Anthracosis is defined as a blackish discoloration of the bronchial mucosa caused by carbon deposition and pigmentation in the airways, leading to bronchial arthrofibrosis. It usually presents in elderly patients with symptoms of chronic cough and dyspnea and is found predominantly in patients with a history of biomass exposure. In addition, a history of exposure to dust and wood smoke is usually present [1]. Although it is predominantly a respiratory disease, cases of systemic involvement presenting with disseminated lymphadenopathy have been infrequently reported [2]. In some studies, it has also been associated with smoking, infectious diseases such as tuberculosis, and malignancies [3,4].

Mediastinal lymphadenopathy can result from a variety of causes, including infections such as tuberculosis, malignancies such as lymphoma or thymoma, and inflammatory disorders such as sarcoidosis, or can sometimes even be benign. Evaluation traditionally involved mediastinoscopy but has recently been superseded by endobronchial ultrasound-guided transbronchial needle aspiration (EBUS-TBNA). F-18-fluorodeoxyglucose positron emission tomography/computed tomography (FDG PET/CT) scans are also currently used to diagnose metastatic disease, but false positives have been known to occur in metabolically active benign diseases that manifest increased FDG uptake, one of which is nodal anthracosis [3,5]. We describe a patient who presented with B-symptoms and diffuse lymphadenopathy, was found to have hypermetabolic mediastinal lymph nodes on an FDG PET/CT scan, and was diagnosed with this infrequently described condition.

## Case presentation

**Figure 1:**
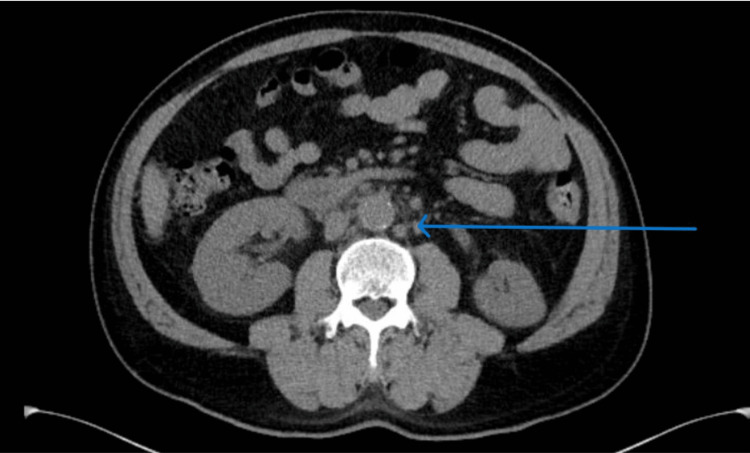
CT scan of the abdomen and pelvis with mesenteric lymphadenopathy. CT: computed tomography.

**Figure 2:**
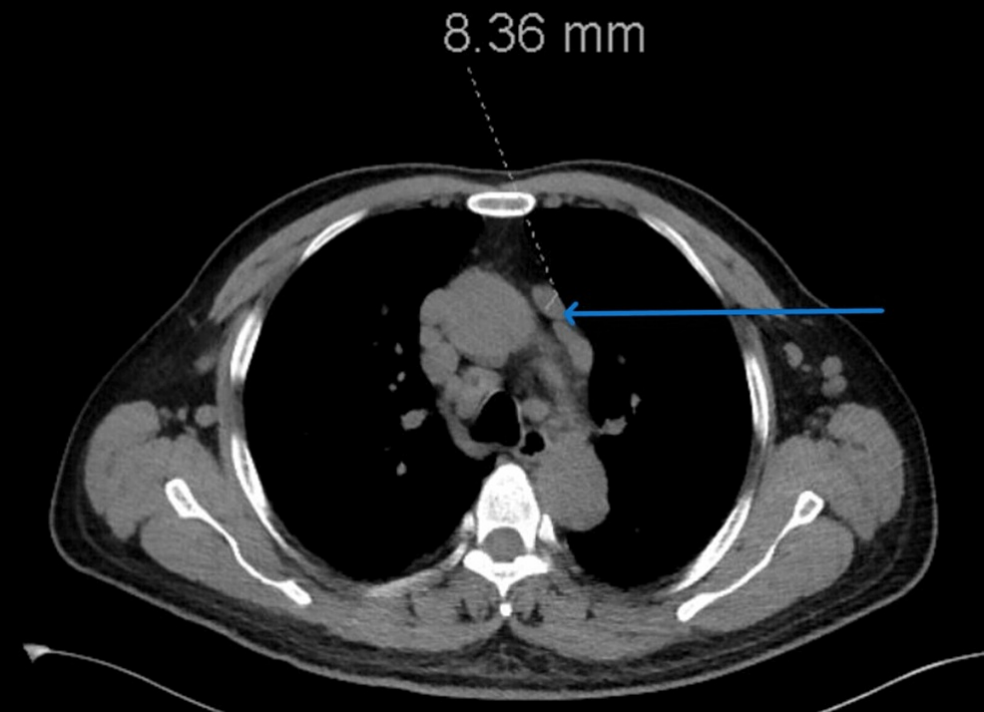
CT scan of the chest with mediastinal lymphadenopathy. CT: computed tomography.

A 64-year-old Vietnamese gentleman presented with complaints of generalized weakness, low-grade fever, night sweats, and unquantifiable weight loss over the past month leading up to hospitalization. He was a former smoker with a 50-pack-year smoking history and had prostate cancer diagnosed three months ago, for which he underwent a radical prostatectomy and was awaiting radiation therapy. He had no other significant history, including tuberculosis or any other malignancies. A staging CT scan had revealed mesenteric lymphadenopathy, for which he was also pending evaluation. He was a retired clerk and denied occupational or environmental exposure to smoke or fumes of any kind. He was hemodynamically stable on admission. An elderly, emaciated gentleman was examined and found to have bilateral firm, non-matted, non-tender inguinal and axillary lymphadenopathy, with the largest measuring 2 cm in diameter and no associated scars, sinuses, or skin changes. Genitourinary examination was normal, without any ulcers or rashes. Laboratory investigations were significant for hemoglobin of 11.3 g/dL, platelets 42000/µL, sodium of 124 mg/dL, erythrocyte sedimentation rate (ESR) 88 mm/h (2-10), C-reactive protein (CRP) 132 mg/L (0-5), lactate dehydrogenase (LDH) 220 U/L (<226), albumin-globulin (A-G) reversal, and sterile blood cultures, with peripheral smear demonstrating anisocytosis and few polychromatic red cells. CT of the chest, abdomen, and pelvis revealed diffuse lymphadenopathy involving the mediastinum, hilar, axillary, mesenteric, retroperitoneal, and bilateral iliac chains with multiple bilateral pulmonary nodules along with inflammatory stranding around the infrarenal aorta (Figures 1 and 2).

**Figure 3:**
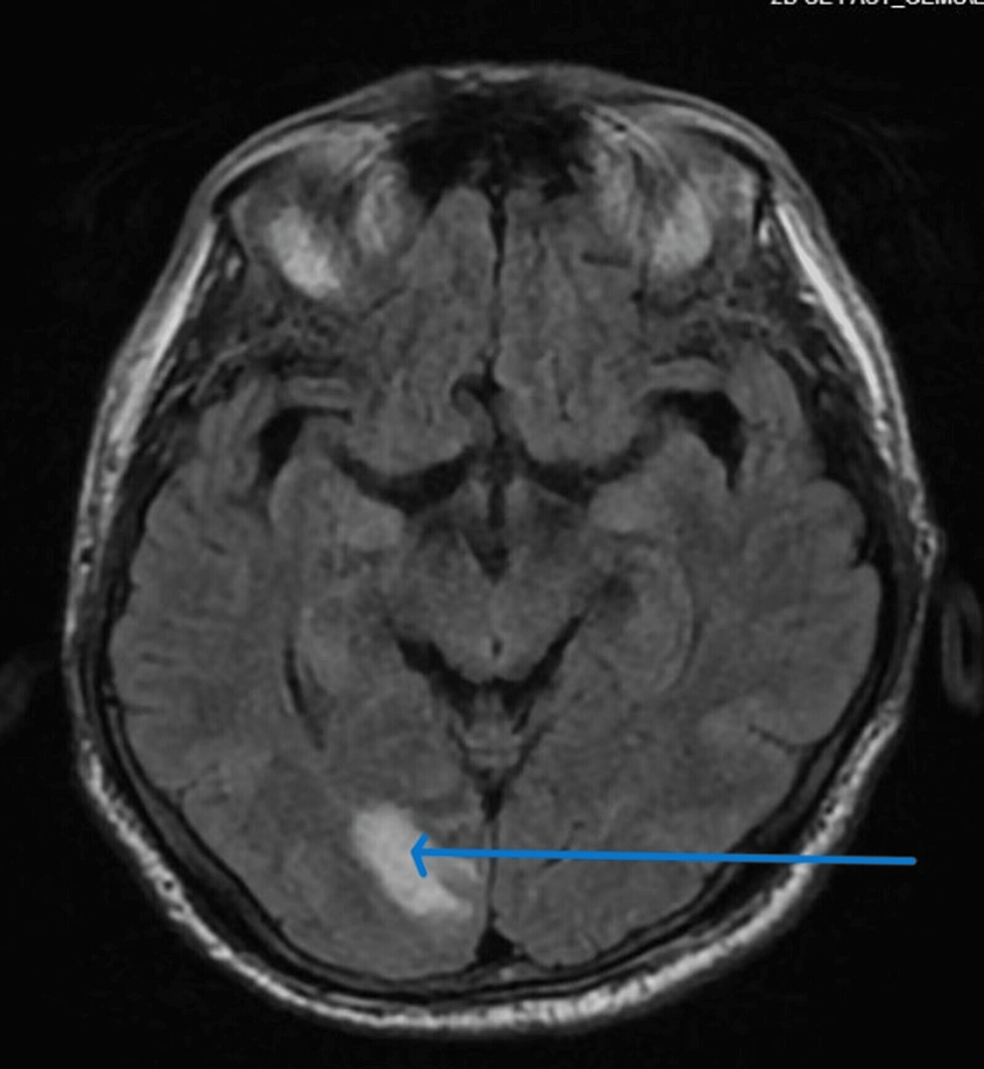
MRI of the brain demonstrating right occipital linear enhancement with surrounding vasogenic edema.

The disseminated intravascular coagulation panel was negative with normal fibrinogen and mildly elevated D-dimer. Autoimmune workup, including antinuclear antibody (ANA), was negative. Infectious workup included *Babesia*, *Ehrlichia*, *Anaplasma*, Lyme serology, QuantiFERON-TB Gold, Epstein-Barr virus (EBV), cytomegalovirus (CMV), herpes simplex virus (HSV), varicella, HIV, and hepatitis panel, all of which were negative. Urine studies revealed features of the syndrome of inappropriate antidiuretic hormone secretion (SIADH) managed with fluid restriction, with which his hyponatremia improved. He was initially managed with broad-spectrum antibiotics, which were discontinued after the negative infectious workup. He also complained of a new-onset holocranial headache post-admission. An examination revealed no neck stiffness or focal neurological deficits. An MRI of the brain with contrast revealed focal right occipital enhancement with vasogenic edema, concerning leptomeningeal involvement, along with occipital lymphadenopathy and a heterogeneously enhancing upper cervical spine due to a possible marrow invasion (Figure 3).

A lumbar puncture was planned to confirm the diagnosis but was deferred at the patient's request. An excisional lymph node biopsy of the left axillary lymph node revealed reactive follicular hyperplasia with no evidence of malignancy, with flow cytometry negative for any evidence of B- or T-cell malignancies. 

**Figure 4:**
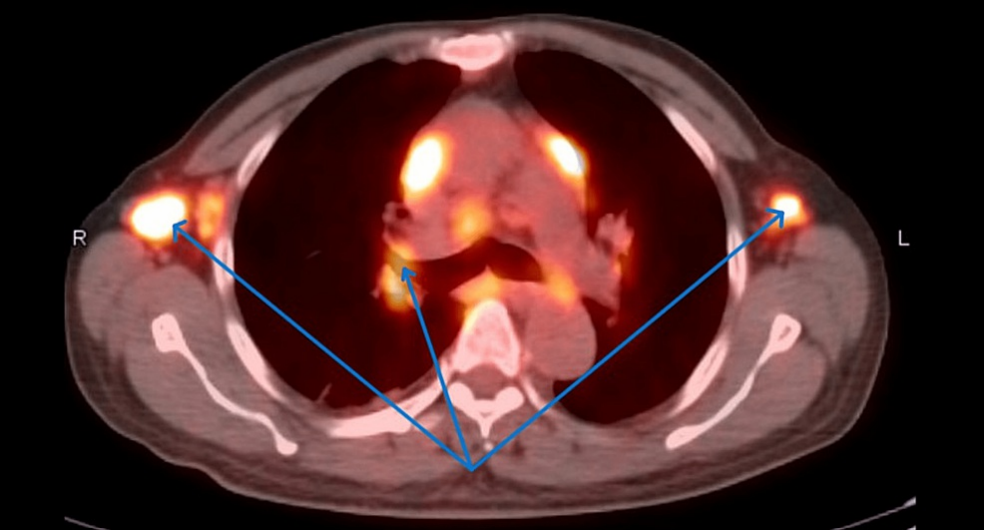
FDG PET/CT showing extensive hypermetabolic disease with bulky mediastinal and axillary lymphadenopathy. FDG PET/CT: F-18-fluorodeoxyglucose positron emission tomography/computed tomography.

**Figure 5:**
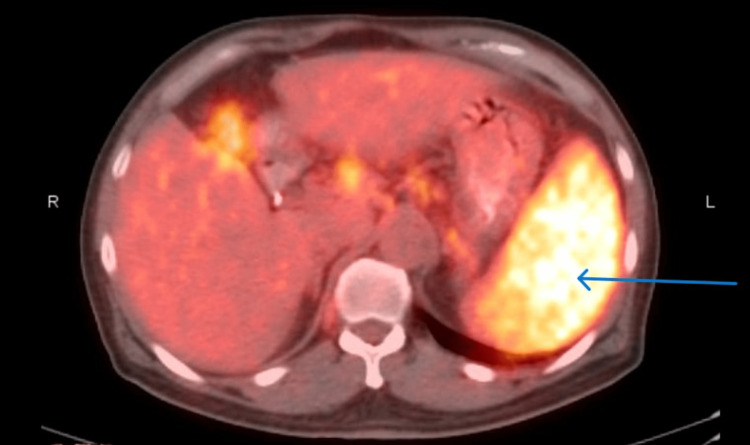
FDG PET/CT showing hypermetabolic spleen. FDG PET/CT: F-18-fluorodeoxyglucose positron emission tomography/computed tomography.

He continued to have persistent low-grade fevers. A bone marrow biopsy showed 70% cellularity with paratrabecular interstitial lymphoid aggregates composed of both T and B cells, which was nonspecific, and flow cytometry could not be done due to dry tap. He finally underwent an FDG PET/CT scan, which showed extensive hypermetabolic disease both above and below the diaphragm with bulky mediastinal adenopathy and splenomegaly (Figures 4 and 5).

**Figure 6:**
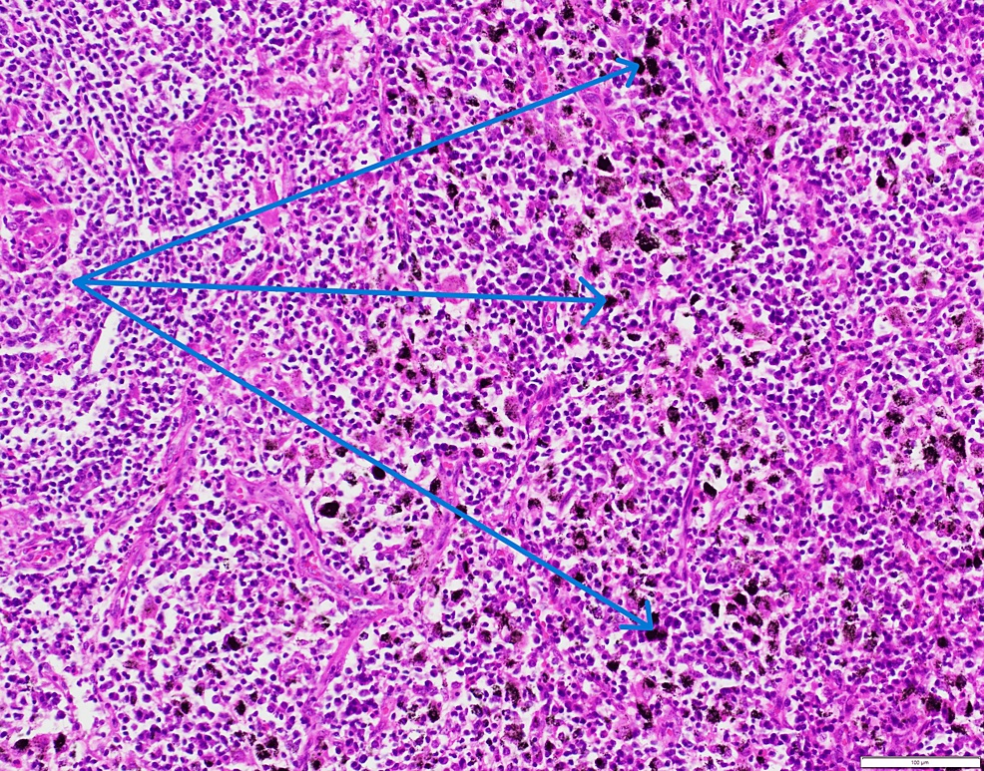
Mediastinal lymph node biopsy demonstrating reactive hyperplasia with anthracitic pigment deposition

Subsequently, he underwent a mediastinoscopy and multiple biopsies of the mediastinal lymph nodes, which demonstrated reactive hyperplasia with abundant anthracitic pigment on microscopic examination, consistent with the diagnosis of nodal anthracosis (Figure 6).

He was managed conservatively and discharged with a plan for outpatient follow-up. On follow-up evaluation six weeks later, his symptoms were found to be improving spontaneously without any intervention. 

## Discussion

Nodal anthracosis with FDG PET/CT-positive mediastinal and hilar lymphadenopathy is considered a rare presentation of this clinical entity that mimics infectious conditions, granulomatous diseases, and malignancies [6,7]. The pathogenesis involves the migration of dust-laden alveolar macrophages to lymph nodes following inhalation exposure and persistent antigenic stimulation, with resultant inflammatory activity highlighted on PET/CT [8]. Most often, patients do not have a classical history of environmental exposure. Pulmonary function tests typically demonstrate an obstructive pattern with no response to bronchodilators and normal diffusing capacity of the lungs for carbon monoxide (DLCO), but a restrictive pattern has also been noted. Histopathology findings include anthracotic granules, inside and outside of macrophages, and edema with scattered inflammatory cells [2].

Anthracosis presenting as FDG PET/CT-positive lymphadenopathy can mimic and often co-exist with other sinister conditions, necessitating clinical and radiological workup to rule out potential underlying etiologies. The key radiographic features that may help to differentiate nodal anthracosis from lymph nodes with the metastatic disease include the smaller size and symmetric bilateral distribution of lymph nodes with anthracosis, as opposed to enlarged and asymmetric in metastasis, but are not always sensitive [9]. Management is usually conservative, with accurate lymph node sampling to confirm the diagnosis and regular follow-up being key aspects.

Our patient denied any significant history of smoke or biomass exposure but did have an extensive smoking history. His B-symptoms and weight loss with lymphadenopathy were initially attributed to a possible infection or malignancy. However, an extensive infectious workup was negative, and a possible hematological malignancy could not be identified despite a bone marrow biopsy and two lymph node biopsies with flow cytometry. The metabolically active FDG-avid lymphadenopathy on the PET scan was hence attributed to nodal anthracosis, which was confirmed on the mediastinal lymph node biopsy.

## Conclusions

Deposition of anthracotic pigment within lymph nodes can often cause persistent inflammatory activity, manifesting as metabolically active pathology such as malignancy or infection on FDG PET-CT. In the case of mediastinal lymphadenopathy, this warrants evaluation with EBUS-TBNA or invasive measures such as mediastinoscopy and biopsy to rule out more sinister pathologies. Nodal anthracosis should be considered an infrequent but important explanation of FDG-positive disseminated lymphadenopathy in patients without known biomass exposure. 
